# The Eps1p Protein Disulfide Isomerase Conserves Classic Thioredoxin Superfamily Amino Acid Motifs but Not Their Functional Geometries

**DOI:** 10.1371/journal.pone.0113431

**Published:** 2014-12-01

**Authors:** Shai Biran, Yair Gat, Deborah Fass

**Affiliations:** Department of Structural Biology, Weizmann Institute of Science, Rehovot, Israel; Yale University School of Medicine, United States of America

## Abstract

The widespread thioredoxin superfamily enzymes typically share the following features: a characteristic α-β fold, the presence of a Cys-X-X-Cys (or Cys-X-X-Ser) redox-active motif, and a proline in the *cis* configuration abutting the redox-active site in the tertiary structure. The Cys-X-X-Cys motif is at the solvent-exposed amino terminus of an α-helix, allowing the first cysteine to engage in nucleophilic attack on substrates, or substrates to attack the Cys-X-X-Cys disulfide, depending on whether the enzyme functions to reduce, isomerize, or oxidize its targets. We report here the X-ray crystal structure of an enzyme that breaks many of our assumptions regarding the sequence-structure relationship of thioredoxin superfamily proteins. The yeast Protein Disulfide Isomerase family member Eps1p has Cys-X-X-Cys motifs and proline residues at the appropriate primary structural positions in its first two predicted thioredoxin-fold domains. However, crystal structures show that the Cys-X-X-Cys of the second domain is buried and that the adjacent proline is in the *trans*, rather than the *cis* isomer. In these configurations, neither the “active-site” disulfide nor the backbone carbonyl preceding the proline is available to interact with substrate. The Eps1p structures thus expand the documented diversity of the PDI oxidoreductase family and demonstrate that conserved sequence motifs in common folds do not guarantee structural or functional conservation.

## Introduction

The Protein Disulfide Isomerase (PDI) family is defined by the presence of at least one thioredoxin-fold (Trx) domain and localization to the secretory pathway [1]. Most PDI proteins contain a redox-active Cys-X-X-Cys motif in one or more of their Trx domains. The yeast endoplasmic reticulum (ER) contains five members of the PDI family, differing widely in amino acid sequence, domain composition, and expression level [2]. Four of these yeast PDI proteins contain Cys-X-X-Cys motifs and thus have the potential to participate in dithiol/disulfide exchange reactions. Such reactions can function to introduce disulfide bonds into substrate proteins during oxidative protein folding or to reduce disulfide bonds, for example as a prelude to extraction of terminally mis-folded substrates from the ER for degradation in the cytosol. Pdi1p is the most highly expressed member of the family and is the only member essential in yeast under standard laboratory conditions [3]. The other PDI proteins may have restricted substrate sets and specialized functions, but these largely remain to be discovered. Exploring the structural and functional diversification in the yeast PDI family is essential for fully characterizing ER function in this model organism, in which many fundamental cell biological processes were first revealed.

One of the yeast PDI family members is Eps1p. Based on amino acid sequence, Eps1p appears to be a four-domain protein with a carboxy-terminal membrane anchor. It contains two Cys-X-X-Cys motifs, one in each of the first two Trx domains. Little is known about the biological function of Eps1p, but secretion of heterologous proteins increases when Eps1p is absent, suggesting a role in protein retention and degradation [4]. Furthermore, *in vitro* assays showed that Eps1p is a poor substrate for the ER sulfhydryl oxidase Ero1p [5], the enzyme that functions as the disulfide donor to Pdi1p to promote oxidative folding of nascent proteins. The second Eps1p Cys-X-X-Cys motif was also found to be extremely resistant to reduction relative to other PDI family Cys-X-X-Cys motifs [5]. It was not known whether this behavior resulted from the local geometry of the disulfide, its chemical environment, or perhaps its burial against another part of the protein. These questions motivated a structural investigation of Eps1p, focusing on the tandem Cys-X-X-Cys domains (Trx1-Trx2).

## Results

### Analysis of Eps1p di-cysteine motifs

**Figure 1:**
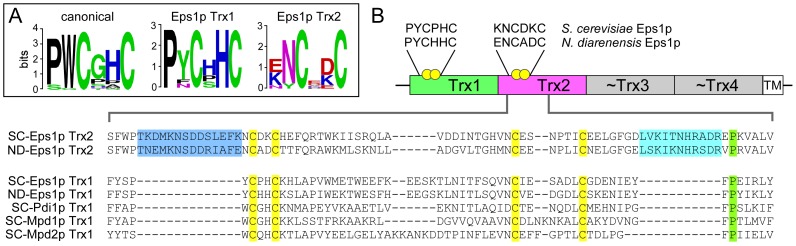
Eps1p amino acid sequence motifs. (A) Active-site amino acid sequence motif logos comprising yeast and human PDI family proteins (canonical) and each of the two Eps1p amino-terminal thioredoxin domains (Eps1p Trx1 and Eps1p Trx2). Yeast species used for Eps1p logos are listed in the Methods. Both Eps1p Cys-X-X-Cys motifs differ from the canonical sequence. (B) Domain map of Eps1p with Cys-X-X-Cys motifs illustrated as paired yellow balls. The motif sequences of Eps1p from the two yeast species used in this study are displayed. Shown below are the sequences of a larger segment of the Trx2 domain from *S. cerevisiae* and *N. dairenensis* Eps1p, compared to homologous segments of other *S. cerevisiae* PDI family proteins. Insertions in the Eps1p Trx2 domain sequence are highlighted in marine blue and cyan.

The Eps1p Cys-X-X-Cys motifs are embedded in unusual amino acid sequence contexts. The most common active-site sequence in the PDI family is Pro-Trp-**Cys**-Gly-His-**Cys** (PW**C**GH**C**), whereas the two di-cysteine motifs of *S. cerevisiae* Eps1p are Pro-Tyr-**Cys**-Pro-His-**Cys** (PY**C**PH**C**) and Lys-Asn-**Cys**-Asp-Lys-**Cys** (KN**C**DK**C**) (Figure 1A). Eps1p is one of the rare PDI family members in yeast and humans lacking a tryptophan residue immediately upstream of the Cys-X-X-Cys motif. In addition, Eps1p differs from other redox-active PDI protein domains by having two insertions of about 13 residues each in its second Trx domain (Figure 1B). One insertion is immediately upstream of the Cys-X-X-Cys motif, and the second precedes a conserved proline found adjacent to the Cys-X-X-Cys in the tertiary structure of Trx domains. These insertions are conserved in Eps1p-like proteins from various budding yeast.

### Crystallization of Eps1p Trx1-Trx2 domains

The first two tandem Trx domains from various Eps1p orthologs were cloned into expression vectors for production in *E. coli*. The Eps1p construct derived from *Naumovozyma dairenensis* (ND-Eps1p) yielded high protein levels and well-diffracting crystals. Diffraction data were collected to 1.8 Å resolution, and the structure was solved by molecular replacement using the amino-terminal domain of *S. cerevisiae* Pdi1p and an isolated Trx β-sheet as search models (see Materials and Methods). The refined ND-Eps1p structure was used in turn to solve the structure of *S. cerevisiae* Esp1p Trx1-Trx2 (SC-Eps1p), crystals of which contained four molecules in the asymmetric unit and diffracted to 2.37 Å resolution. Data collection and refinement statistics are presented in Table 1.

**Table 1:** Data collection and refinement statistics.

	DN-Eps1p	SC-Eps1p
**Data collection**		
Space group	P2_1_2_1_2_1_	P2_1_
Cell dimensions		
*a*, *b*, *c* (Å)	42.68, 63.58, 119.21	40.02 133.03 124.40
α, β, γ (°)	90, 90, 90	90, 92.0, 90
Resolution (Å)	40.2–1.8 (1.86–1.80)*	41.77–2.77 (2.45–2.37)
*R*_meas_ †	0.166 (0.411)	0.166 (0.647)
*I/* *σI*	9.8 (2.9)	8.7 (2.9)
Completeness (%)	97.8 (92.8)	99.4 (99.0)
Redundancy	8.2 (8.1)	4.9 (4.5)
**Refinement**		
Resolution (Å)	40.2–1.8	41.8–2.37
Number of reflections/test set	30,737/2152	52,451/3672
*R*_work_/*R*_free_	0.173/0.212	0.212/0.272
Number of atoms		
Protein	2131	8579
Water	351	240
RMSD		
Bond lengths (Å)	0.007	0.010
Bond angles (°)	0.98	1.28

*Values in parentheses are for the highest resolution shell.

† R_meas_ is as defined in [26].

**Figure 2:**
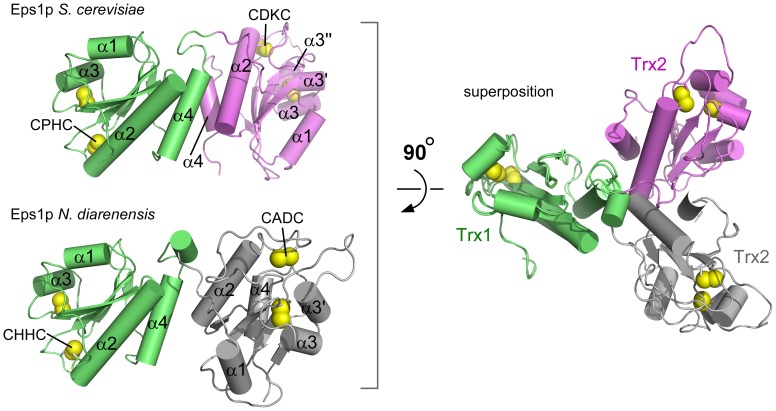
Structures of tandem Trx domains from *S. cerevisiae* and *N. dairenensis* Eps1p. Trx1 domains in the two orthologous structures are shown in green. Trx2 domains are violet or gray. On the left, α-helices are numbered according to their order in the primary structure of each domain. On the right, the two orthologous structures are superposed according to a least-squares fit of the Trx1 domains, illustrating the divergence in relative orientation of the Trx2 domains.

The orientation of the two Trx domains in the ND-Eps1p structure places the two Cys-X-X-Cys motifs at nearly maximal separation, almost 55 Å from one another (Figure 2). The interface between the domains is composed of the last helix in Trx1 (α4) and the carboxy-terminal ends of the active-site helices (α2) of Trx1 and Trx2, burying about 880 Å^2^. The amino acids at the interface, which make hydrophobic and hydrogen bonding interactions, are not notably conserved. Indeed, the SC-Eps1p structure revealed a different orientation between domains and a different interdomain interface (Figure 2). The interface in SC-Eps1p appears to consist primarily of salt bridges, between helix α4 of the Trx1 domain and helices α2 and α4 of the Trx2 domain, burying about 990 Å^2^. The distance between the two Cys-X-X-Cys motifs is about 46 Å in the SC-Eps1p structure. The relative domain orientation is the same in all four copies of SC-Eps1p in the asymmetric unit. Electrostatic surface representations of the Eps1p structures are presented in Figure S1.

### The Eps1p Trx1 domain is similar to canonical PDI domains

**Figure 3:**
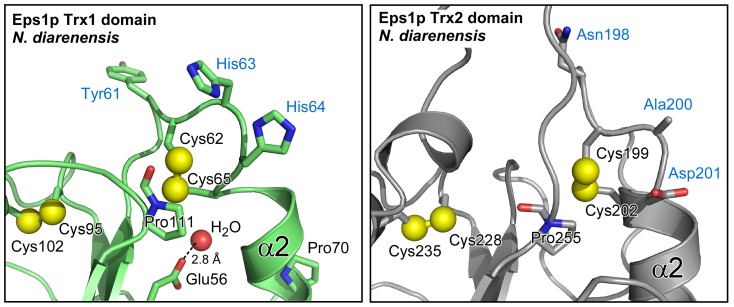
Canonical and non-canonical features of Eps1p. Structure and residue numbering correspond to *N. dairenensis* Eps1p. Blue labels indicate amino acids unique to Eps1p at these positions. Black labels indicate amino acids that are common to many redox-active PDI family Trx domains. Cysteine sulfurs and the oxygen of the water molecule bound by Glu56 in Trx1 are shown at half their van der Waals radii.

The Trx1 domain of Eps1p shows strong overall structural similarity to many other PDI family Trx domains. A particularly close match, with a root mean square deviation (RMSD) of 1.5 Å for 107 Cα positions, was observed between the ND-Eps1p Trx1 domain and the third domain of ERp46 (3UVT) [6]. A RMSD of 2.2 Å over 108 residues was observed compared to the fourth domain (the **a′** domain) of Pdi1p (2B5E) [7]. The Eps1p Trx1 domain has familiar structural and functional features observed previously in PDI domains, including a proline five residues downstream of the second cysteine in the Cys-X-X-Cys motif, a buried glutamic acid probably involved in deprotonating the second Cys-X-X-Cys cysteine *via* an intermediary water molecule [8],[9], and the *cis*-proline spatially adjacent to the Cys-X-X-Cys disulfide bond [10] (Figure 3). These features are major contributors to the dithiol/disulfide exchange mechanism, and their presence suggests that the Eps1p Trx1 domain is catalytically active.

The most notable deviation of the ND-Eps1p Trx1 domain from other PDI domain structures is an unwinding of the first turn of the Cys-X-X-Cys helix, such that the backbone carbonyls of the first three residues in the motif are not properly hydrogen-bonded to the remainder of the helix. This deviation from canonical Trx structure may be due to crystal packing, as the Trx1 Cys-X-X-Cys motif (*i.e*., Cys-His-His-Cys) is intimately involved in a crystal contact mediated by the two histidines, and SC-Eps1p does not exhibit the same phenomenon.

### The Eps1p Trx2 domain is divergent from both redox-active and -inactive PDI domains

**Figure 4:**
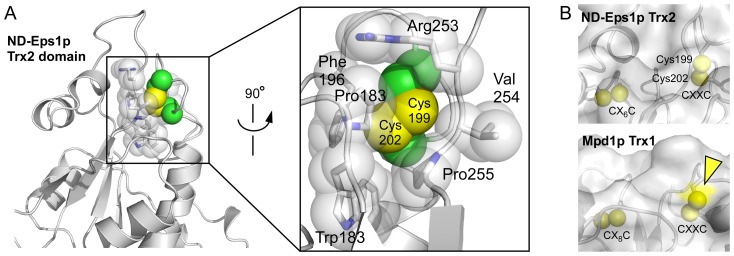
Buried Cys-X-X-Cys of Eps1p Trx2 domain. (A) A segment of the *N. dairenensis* Eps1p (ND-Eps1p) Trx2 domain is shown as a ribbon diagram, highlighting amino acid side chains that form the hydrophobic pocket for the Cys-X-X-Cys motif (Cys199-Cys202). Space-filling representation of these side chains emphasizes the tight packing and solvent exclusion provided by these residues. Cysteine sulfurs are shown as yellow van der Waals spheres. Cysteine Cα and Cβ atoms are green. (B) The Eps1p Trx2 cysteines are buried below the gray surface. In the Mpd1p panel, the yellow arrowhead points out surface exposure (bright yellow) of the amino-terminal Cys-X-X-Cys cysteine. Sulfurs are shown at 0.6 of their van der Waals radii.

In contrast to the Trx1 domain, the functional capabilities of the Eps1p Trx2 domain are less clear from the structure. Unlike the non-catalytic Trx domains found in many PDI family proteins (known as “**b**” domains), the Eps1p Trx2 domain contains both a Cys-X-X-Cys motif and an additional, “structural” disulfide characteristic of redox-active PDI Trx domains (known as “**a**” domains) [11],[12]. In addition, the Eps1p Trx2 domain has a proline (Pro255; numbering hereafter is for the full-length *N. dairenensis* Eps1p sequence) in the same position in the tertiary structure as the *cis*-proline common to Trx domains (Figure 3). However, Eps1p Trx2 lacks many features typical of redox-active domains and contains additional features not usually seen in those domains. For example, there is no buried water molecule or acidic residue at the appropriate place to participate in proton transfer from the resolving cysteine in a dithiol/disulfide exchange reaction. Instead, the environment of the Cys-X-X-Cys disulfide is hydrophobic, consisting in ND-Eps1p of two proline side chains, a phenylalanine, a tryptophan, and the aliphatic portion of an arginine side chain (Figure 4). In addition, the proline juxtaposed to the Cys-X-X-Cys motif is found in the *trans*, not *cis*, configuration (Figure 3) in the crystal structures of both Eps1p orthologs. Consequently, the backbone carbonyl of the peptide preceding the proline is not oriented for hydrogen bonding with an incoming substrate.

**Figure 5:**
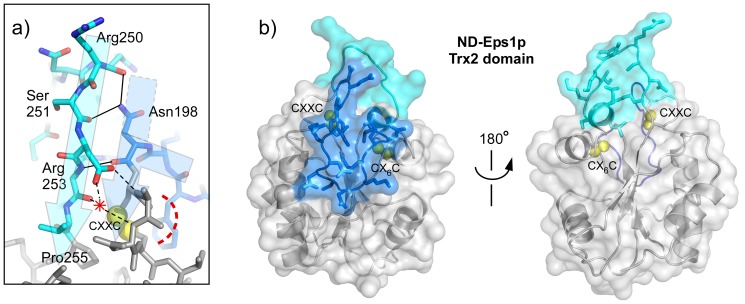
Insertions in the Trx2 domain. (A) A section of the interface between the two insertions in Trx2 is shown to illustrate the hydrogen bonds (solid black lines) that support extension of the penultimate β-strand outward from the domain. Dashed lines show the hydrogen bonds made by the Cyx-X-X-Cys backbone N-H groups. The position of a water molecule is indicated by a red asterisk. Yellow spheres at half their van der Waals radii correspond to the Cys-X-X-Cys cysteine sulfurs. Semi-transparent arrows highlight the approximate paths of the polypeptide backbone in the two loops. The dashed tail of the right, kinked arrow highlights Asn198 and its role in providing β sheet-like hydrogen bonding to the cyan insertion. The red dashed arc emphasizes lack of hydrogen bonding of the Cys-X-X-Cys motif carbonyls to the downstream helix. (B) Insertions are shown in the context of the entire ND-Eps1p Trx2 domain and colored according to Figure 1 and panel A, with the amino-terminal insertion in marine blue and the carboxy-terminal insertion in cyan. Sulfurs are shown at 0.6 of their van der Waals radii.

In fact, incoming substrate would have difficulty accessing the Trx2 Cys-X-X-Cys disulfide as observed in both orthologous Eps1p structures. Unlike other PDI Cys-X-X-Cys motifs, which expose their backbone N-H groups to solvent, the N-H groups of the Eps1p Trx2 Cys-X-X-Cys motif are hydrogen-bonded to neighboring regions of the protein (Figure 5A, dashed lines). The N-H group of the first “X” position interacts with a nearby aspartate side chain (Asp252), while the N-H group of the second “X” appears to form water-bridged hydrogen bonds with the same aspartate and with the backbone carbonyl of the amino acid residue following the aspartate. Furthermore, in Eps1p Trx2, the first two or three backbone carbonyls in the Cys-X-X-Cys motif are not directly hydrogen bonded to the downstream helix, allowing the motif to tilt inwards toward the domain core. The Cys-X-X-Cys disulfide is thus effectively sealed off from solution.

Further obscuring the Trx2 active site in Eps1p, the two loop insertions are draped over the end of the domain containing the Cys-X-X-Cys motif (Figure 5B). The second insertion, upstream of the *trans*-proline, forms a pseudo-β-sheet interaction with the polypeptide segment upstream of the Cys-X-X-Cys. Specifically, the backbone N-H of the arginine (Arg253) two residues before the *trans*-proline (Pro255) is in position to hydrogen bond to the backbone C = O of the asparagine (Asn198) preceding the first Cys-X-X-Cys cysteine (Figure 5A, solid lines). The C = O of the serine (Ser251) four residues before the *trans*-proline hydrogen bonds to the side-chain N-H of Asn198, which mimics a hydrogen bond acceptor in a β-sheet while the main chain kinks at this point. These extended pseudo-β-sheet interactions greatly elongate the penultimate β-strand in the Trx2 domain; the amino acid segment from Arg250 to Asn263 spans about 45 Å, in contrast to the corresponding strand in other PDI family Trx domains, which typically extends less than 30 Å across the compact, globular domain.

## Discussion

When the structure of thioredoxin was first reported in 1975, it was described as a “male” enzyme because the Cys-X-X-Cys active-site motif projects into solvent rather than being at the base of a surface pocket as in other enzymes [13]. Since then, high-resolution structures of hundreds of Trx proteins and domains have revealed the Cys-X-X-Cys motif to be at the solvent-exposed amino-terminus of a helix. The discovery of a Trx domain that retains classic motifs of redox-active members of the superfamily but employs them differently is therefore noteworthy. The Trx2 domain of Eps1p has hidden its “masculinity” due to burial of the Cys-X-X-Cys in the hydrophobic core, facilitated by rearrangements near the amino terminus of helix α2 and by two sequence insertions in the domain. The Cys-X-X-Cys and the adjacent proline of the Eps1p Trx2 domain appear to have evolved from functional into structural features. The conserved amino acids roughly retain their positions in the domain fold but assume locally distinct configurations that dramatically alter their accessibility and potential functionality.

A more detailed analysis of the geometry of the Trx2 Cys-X-X-Cys motif in Eps1p allows comparison with canonical redox-active Trx Cys-X-X-Cys motifs. Functional Trx motifs are typically of the +/−RHHook designation [14]. Table 2 shows the side chain dihedral angles and distances between the cysteine Cα atoms for the known yeast PDI family structures: Pdi1p, Mpd1p, and Eps1p. Pdi1p was observed in a mixture of redox states [7], but the Mpd1p Cys-X-X-Cys [15] can be considered an example of a well-formed, oxidized PDI-family +/−RHHook, except that the Cα-Cα distance between its cysteines is shorter than that reported for most +/−RHHook disulfides [14]. In comparison to Mpd1p, the Eps1p Trx2 Cys-X-X-Cys disulfide deviates mainly in the Χ1 and Χ2 side chain dihedral angles. Eps1p misses the +/−RHHook classification due to a change in the first cysteine Χ1 angle of about 50°. In addition, Χ2 of the first cysteine and X1 of the second cysteine are both about 30° more negative than in the Mpd1p Trx1 Cys-X-X-Cys. Another observation regarding the Eps1p Trx2 domain is that the dihedral angles of its Cys-X-X-Cys are relatively uniform in the four molecules in the asymmetric unit of the SC-Eps1p crystals. In contrast, the SC-Eps1p Trx1 Cys-X-X-Cys dihedral angles show greater deviations among the multiple copies of the protein structure. This observation is consistent with the burial of the Trx2 Cys-X-X-Cys motif and its lower temperature factors in all four molecules. In ND-Eps1p, the temperature factors of the Trx2 Cys-X-X-Cys motif are also slightly lower than those of the Trx1 motif, even though the latter is stabilized by a crystal contact.

**Table 2:** PDI protein disulfide classifications.

	Dihedral angle					
	Χ1 (°)	Χ2 (°)	Χ3 (°)	Χ2′ (°)	Χ1′ (°)	Cα-Cα (Å)
SC-Pdi1p Trx1 (a) (ox)	157.2	-124.1	72.2	82.6	−57.6	5.13
SC-Pdi1p Trx4 (a′) (red)	177.4	N/A†	N/A	N/A	−67.8	5.58
SC-Mpd1p Trx1 chain A	136.4	−117.2	89.0	75.2	−56.9	5.22
SC-Mpd1p Trx1 chain B	139.6	−119.1	87.3	74.9	−56.1	5.16
ND-Eps1p Trx1*	165.7	−159.8	83.9	74.4	−70.4	5.62
ND-Eps1p Trx2	−171.5	−146.8	83.5	73.4	−92.7	5.39
SC-Eps1p Trx1 chain A	174.0	−151.9	73.3	60.7	−37.9	5.21
SC-Eps1p Trx1 chain B	171.3	−144.8	61.2	74.7	−45.2	5.00
SC-Eps1p Trx1 chain C	166.5	−139.6	59.7	71.8	−38.8	5.10
SC-Eps1p Trx1 chain D	168.1	−132.5	74.1	57.8	−36.2	5.39
SC-Eps1p Trx2 chain A	−176.5	−151.0	97.4	64.9	−83.4	5.49
SC-Eps1p Trx2 chain B	180.0	−147.0	91.9	72.4	−83.8	5.65
SC-Eps1p Trx2 chain C	179.8	−144.4	91.5	72.9	−84.3	5.65
SC-Eps1p Trx2 chain D	−177.4	−144.2	89.1	66.8	−82.0	5.53

*The Trx1 Cys-X-X-Cys of ND-Eps1p is involved in a crystal contact.

† “N/A” stands for “not applicable.” In the absence of a disulfide bond, there are no Χ2 or Χ3 dihedral angles.

The non-canonical geometry and environment observed for the Trx2 Cys-X-X-Cys in the Eps1p structures explains previous observations regarding the reactivity of this domain with reducing agents. In a study reporting the reduction-oxidation potentials of yeast PDI family proteins, it was noted that the Trx2 domain of *S. cerevisiae* Eps1p was particularly resistant to reduction [5]. A high concentration of dithiothreitol (DTT) failed to appreciably reduce the Trx2 Cys-X-X-Cys motif, unless denaturing detergent was added to disrupt the tertiary structure. The poor solvent-accessibility of the Trx2 Cys-X-X-Cys motif seen in the crystal structures and its low temperature factors suggests that Eps1p, in isolation and in physiological buffer, does not readily sample conformations in which reducing agents can attack the Trx2 disulfide.

An interesting question that then arises is whether a conformational change might expose and activate the buried Eps1p Trx2 Cyx-X-X-Cys. Using a similar strategy as that used to demonstrate a pH-mediated change in thiol exposure in the human PDI protein ERp44, which is involved in a Golgi-to-ER cysteine-based protein retrieval mechanism [16], we tested, in the context of the nearly full-length *S. cerevisiae* Eps1p truncated at the transmembrane region, whether pH (6.0 to 7.5) triggers a change in the reactivity of the Trx2 Cys-X-X-Cys motif (see Methods). No change was observed for pH treatment (data not shown). We did observe increased reactivity of the Trx Cys-X-X-Cys motif as the temperature was increased above 35°C, which may correspond to global unfolding of the protein (Figure S2). Nevertheless, other, more subtle, mechanisms for perturbing Eps1p and exposing the buried Cys-X-X-Cys may exist and be physiologically relevant. Such mechanisms may include binding to a partner or substrate protein or association with the ER membrane. It is worth restating, however, that all four molecules in the asymmetric unit of the SC-Eps1p crystals show the same conformation, with the Cys-X-X-Cys motif buried, suggesting that this structure predominates for the two-domain fragment in solution at 20°C.

With structures of the Cys-X-X-Cys-containing Trx domains of three of the five yeast PDI family proteins determined so far, the exploration of ER oxidoreductases in this organism is advancing. This handful of yeast PDI proteins shows remarkable diversity in domain composition, redox properties, and reactivity with other ER enzymes [5],[7],[15]. Yeast PDI diversification serves as a model for evolution of the family in other organisms, such as humans, which contain more than 20 members. We are beginning to understand how structural features influence redox activity, but it remains to be determined how redox activity relates to the substrate set and physiological activities of non-essential yeast PDI proteins.

## Materials and Methods

### Amino acid sequence analysis

Eps1p logos were made using the Trx1 and Trx2 sequences of the Eps1p orthologs from the following yeast species: *Saccharomyces cerevisiae, Naumovozyma dairinensis, Saccharomyces arboricola, Lachancea thermotolerance, Torulaspora delbrueckii, Zygosaccharomyces rouxii, Kluyveromyces lactis, Candida glabrata, Tetrapisispora phaffii, Eremothecium cymbalariae.* The crystallized proteins correspond to the NCBI reference amino acid sequences: SC-Eps1p, NP_012261.1; ND-Eps1p, XP_003668384.1.

### Plasmid construction, protein expression, and protein purification

The sequence coding for *S. cerevisiae* Eps1p (residues 28–295), was inserted into the pET-15b vector (Novagen) between the NdeI and BamHI restriction sites. The sequence coding for *N. dairenensis* Eps1p (residues 32–294), codon-optimized for expression in *E. coli* (Genscript), was inserted by restriction-free cloning into pET-15b immediately downstream of the thrombin cleavage site. Proteins were expressed in the *E. coli* strain Origami B (DE3) (Novagen). Cells containing the expression plasmid were grown to OD_595nm_ of 0.6 at 37°C, isopropyl-1-thio-β-D-galactopyranoside was added to a final concentration of 0.5 mM, and the cultures were grown at 15°C for a further 40 h before harvesting. Cell pellets were suspended in 20 mM sodium phosphate buffer, pH 7.6, 500 mM NaCl, and 20 mM imidazole, sonicated, and centrifuged for 30 min at 40,000g to remove cell debris. Proteins were purified by Ni-NTA chromatography (GE-Healthcare). Eluted ND-Eps1p was concentrated and exchanged into 10 mM Tris buffer, pH 7.6, 100 mM NaCl on a PD-10 desalting column, and further purification was performed by size exclusion chromatography in the same buffer. Peak elution fractions were pooled, and the protein was concentrated to 12 mg/ml. SC-Eps1p eluted from the Ni-NTA column was exchanged into 20 mM sodium phosphate, pH 6.8, 100 mM NaCl, 10 mM imidazole and subjected to cleavage by thrombin. Protein was re-applied to Ni-NTA, and unbound material was collected and further purified by gel filtration in 10 mM Tris buffer, pH 7.6, 100 mM NaCl. SC-Eps1p peak fractions were pooled and concentrated to 12 mg/ml.

### Crystallization, data collection, and structure solution

Crystallization conditions were screened at the Israel Structural Proteomics Center and optimized using hanging- or sitting-drop vapor-diffusion at 20°C. The ND-Eps1p stock solution (1.5 µl) was mixed with well solution (1.5 µl) containing 25% w/v polyethylene glycol (PEG) 8,000 Da, 0.1 M MES buffer, pH 6.0, and 0.2 M calcium acetate. Crystals grown in these conditions were used for streak-seeding sitting drops prepared against a well solution of 30% w/v PEG 8,000 Da, 0.1 M MES buffer, pH 6.0, 80 mM calcium acetate. Crystals were transferred to a solution containing 60% well solution and 25% ethylene glycol and flash frozen in liquid nitrogen. SC-Eps1p stock solution (1 µl) was mixed with well solution (1 µl) containing 24% PEG 4,000 Da, 100 mM sodium acetate, pH 6.4, 25 mM sodium citrate, pH 6.4, and 17% glycerol. These conditions produced clusters of fine plates. Repeated microseeding was used to improve crystal quality, resulting in a small number of small single plates. SC-Eps1p crystals were transferred to a solution containing 25% PEG 4,000 Da, 100 mM sodium acetate, pH 6.4, 25 mM sodium citrate, pH 6.4, 25% glycerol and flash frozen under liquid nitrogen. The vast majority of SC-Eps1p crystals showed severe lattice defects and unusable diffraction. Upon extensive screening, one crystal was found that yielded satisfactory data.

Data for SC-Eps1p were collected to 2.37 Å resolution at beamline ID29 of the European Synchrotron Radiation Facility (ESRF), Grenoble, France, using a Pilatus 6M-F detector. The crystal space group was P2_1_ with unit cell dimensions a = 40.026 Å, b = 133.031 Å, c = 124.406 Å, α = 90°, β = 92°, γ = 90°. Data were processed and scaled using the XDS and XSCALE software packages [17]. Data for ND-Eps1p were collected to 1.8 Å resolution at ESRF beamline ID23-1 using a Pilatus 6M-F detector. The space group was P2_1_2_1_2_1_ with unit cell dimensions a = 42.68 Å, b = 63.58 Å, c = 119.21 Å, α = β = γ = 90°. Data were processed and scaled using HKL2000 [18].

The ND-Eps1p structure was determined by molecular replacement using Phaser [19]. First, an edited structure of the first domain of yeast Pdi1p (pdb ID code 2B5E, residues 22–139) was used to search for the first Trx domain, and suitable rotation and translation solutions were found. The search model for the second Eps1p domain consisted of the β-sheet region (residues 45–47, 63–69, 97–103, 123–127, 137–138) from the human QSOX1 enzyme (pdb ID code 3T59). QSOX1 was selected as the source of the β-sheet because the Phyre2 fold-recognition server [20] returned QSOX1 as the highest scoring template for Eps1p Trx2. After positioning of this β-sheet and calculation of electron density maps, helices and loops were added using Coot [21]. Refinement was done in Phenix [22], and validation of the structure was performed using MolProbity [23], according to which there were no Ramachandran outliers and the structure model was rated in the 99^th^ percentile in its resolution range.

The SC-Eps1p structure was solved by molecular replacement using the individual Trx1 and Trx2 domains of the ND-Eps1p structure. Model rebuilding and refinement were done as for ND-Eps1p. Structure assessment using MolProbity [23] showed no Ramachandran outliers. The SC-Eps1p structure model was rated in the 100^th^ percentile in its resolution range.

Comparison to previously determined protein structures was done using the Dali server [24].

### Thermal denaturation and pH scan experiments

To investigate the effect of pH on the sensitivity of the Eps1p Trx2 domain to reductants, 15 µM recombinant *S. cerevisiae* Eps1p lacking the transmembrane region and with the CXXC motif of the first Trx domain mutated to AXXA [5] was incubated at room temperature for 30 min in 1 mM DTT, 150 mM NaCl, and 100 mM phosphate buffer at pH values ranging from 6.0 to 7.5. As a positive control, one sample was incubated at pH 7.5 with 1 mM DTT and 2% SDS to denature the protein and expose disulfides to reduction. At the end of the incubation, trichloroacetic acid was added to 20%, and the solution was incubated overnight at 4°C. The insoluble material was spun down for 15 min at 14,000 rpm in a microfuge, and the supernatant was aspirated off. Two washes were performed in which 500 µl cold acetone was added, the tubes were centrifuged for 5 min, and the acetone was aspirated off. After the second wash, the tubes were left open to dry for a few minutes, and then 5 µl 10 mM maleimide-functionalized polyethylene glycol (PEG) 5 kD was added, followed by 15 µl SDS-PAGE loading buffer containing 200 mM Tris, pH 6.8, 2% SDS, 20% glycerol, and bromophenol blue. Samples were applied to SDS-PAGE, and proteins were visualized with Coomassie blue stain. The same PEG labeling procedure was applied to Eps1p Trx1-AXXA samples after incubation for 25 minutes at various temperatures, as indicated in Figure S2.

Unfolding of the Trx1-AXXA variant of *S. cerevisiae* Eps1p was assessed by scanning fluorimetry [25]. Melting curves were measured on 15 µM Eps1p in 100 mM sodium phosphate buffer, pH 7.0, 150 mM NaCl, and 20X sypro orange dye (Sigma-Aldrich S5692, supplied as 5000X), with or without 1 mM DTT. Pdi1p and Mpd1p were measured at 16.5 µM and 8.5 µM, respectively, in the same buffer. The experiments were performed in an Applied Biosystems StepOne real-time PCR machine by reading fluorescence in 0.5°C intervals using a temperature gradient moving from 25°C to 95°C at a rate of about 1.6°C/min.

### PDB accession codes

The coordinates and structure factors have been deposited in the Protein Data Bank with codes 4TVE (Nd-Eps1p) and 4TW5 (Sc-Eps1p).

## Supporting Information

Figure S1 Electrostatic surface representations of Eps1p. For orientation, ribbon diagrams of ND-Eps1p and SC-Eps1p are presented above each electrostatic surface representation, which were calculated using the APBS plugin in Pymol^*^ and visualized at threshold +/− 5 k_B_T/e. ^*^Baker NA, Sept D, Joseph S, Holst MJ, McCammon JA. Electrostatics of nanosystems: application to microtubules and the ribosome. Proc. Natl. Acad. Sci. USA 2001;98: 10037–41.
(DOCX)

Figure S2 Thermal unfolding and cysteine exposure in *S. cerevisiae* Eps1p. (A) A fluorescence-based thermal shift assay was used to characterize the non-equilibrium unfolding of Eps1p. The transition midpoint of the thermal unfolding of the Eps1p mutant was 39.5°C with or without 1 mM DTT in solution. For comparison, when yeast Pdi1p and Mpd1p were subjected to the same protocol, both proteins showed a slight increase in thermal stability when their redox-active sites were reduced by DTT, as expected for proteins that function to oxidize substrate cysteines. (B) The Trx1-AXXA variant of Eps1p was incubated at the indicated temperatures for 25 min, precipitated with trichloroacetic acid, and treated with maleimide-derivatized polyethylene glycol of molecular weight 5 kD (mal-PEG). Each mal-PEG addition causes a decrease in migration in SDS-PAGE corresponding to about 15 kD of protein, consistent with all six cysteines in the Eps1p variant being modified. Exposure of the Trx2 CXXC cysteines to reduction by DTT thus correlated with reduction of the two CX_6_C structural disulfides in the Trx1 and Trx2 domains.
(DOCX)
