# Supplementary figures and images for: The Eps1p Protein Disulfide Isomerase Conserves Classic Thioredoxin Superfamily Amino Acid Motifs but Not Their Functional Geometries
Source: PLoS One. 2014 Dec 1;9(12):e113431. doi: 10.1371/journal.pone.0113431 (PMC4249923; doi:10.1371/journal.pone.0113431)

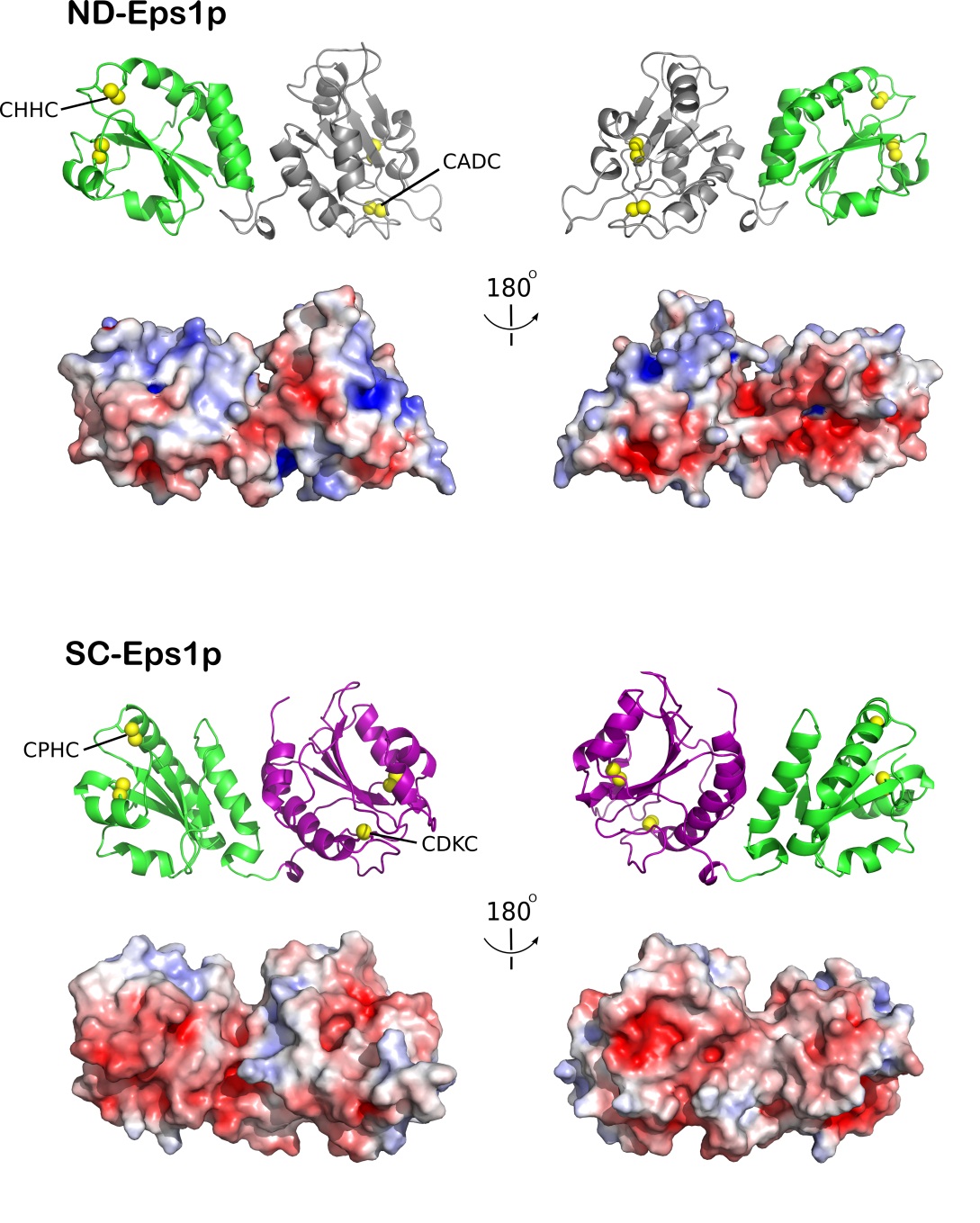

Supplement: Figure S1 — Electrostatic surface representations of Eps1p. For orientation, ribbon diagrams of ND-Eps1p and SC-Eps1p are presented above each electrostatic surface representation, which were calculated using the APBS plugin in Pymol* and visualized at threshold +/− 5 kBT/e. *Baker NA, Sept D, Joseph S, Holst MJ, McCammon JA. Electrostatics of nanosystems: application to microtubules and the ribosome. Proc. Natl. Acad. Sci. USA 2001;98: 10037–41. (DOCX) [file pone.0113431.s001.docx]

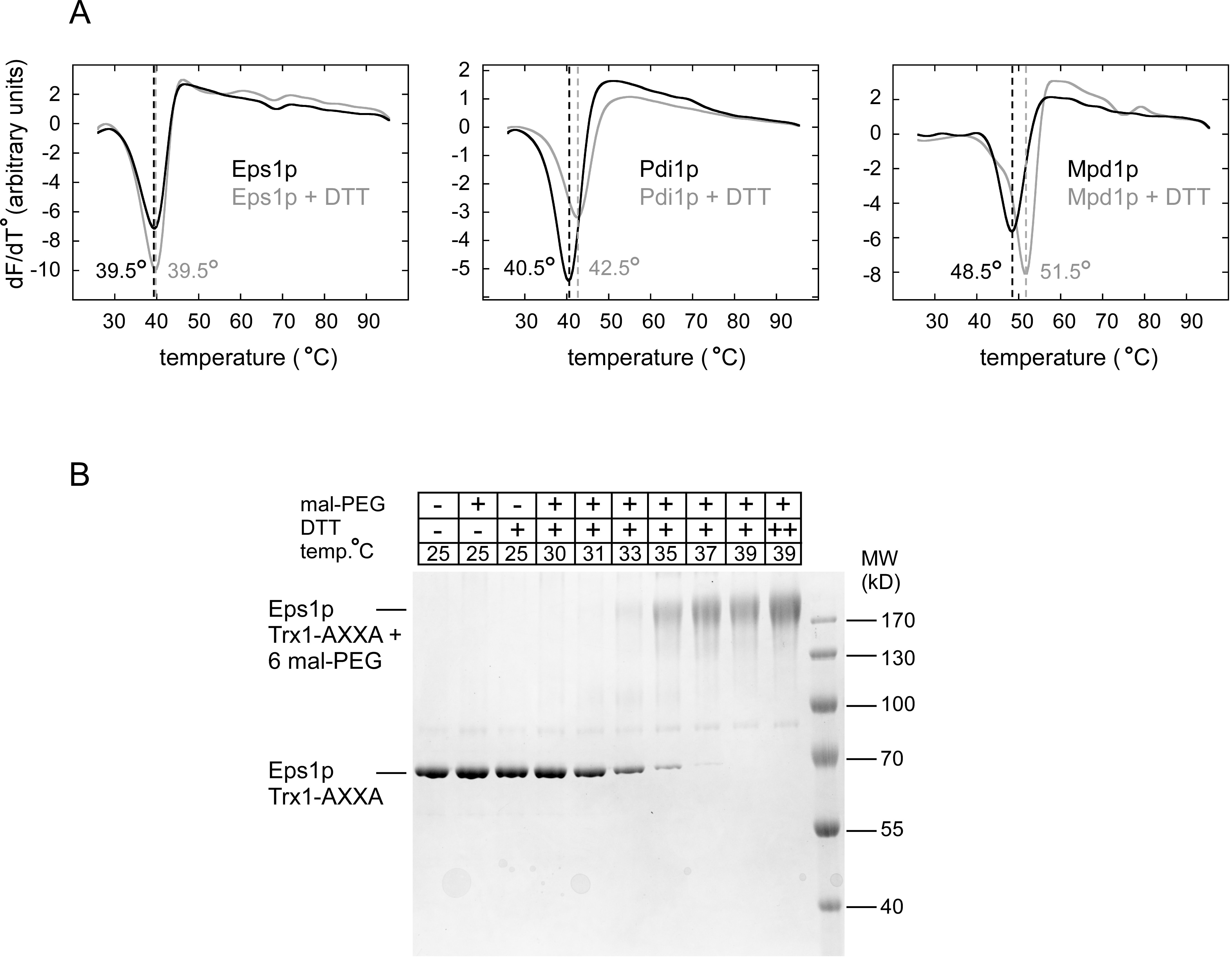

Supplement: Figure S2 — Thermal unfolding and cysteine exposure in S. cerevisiae Eps1p. (A) A fluorescence-based thermal shift assay was used to characterize the non-equilibrium unfolding of Eps1p. The transition midpoint of the thermal unfolding of the Eps1p mutant was 39.5°C with or without 1 mM DTT in solution. For comparison, when yeast Pdi1p and Mpd1p were subjected to the same protocol, both proteins showed a slight increase in thermal stability when their redox-active sites were reduced by DTT, as expected for proteins that function to oxidize substrate cysteines. (B) The Trx1-AXXA variant of Eps1p was incubated at the indicated temperatures for 25 min, precipitated with trichloroacetic acid, and treated with maleimide-derivatized polyethylene glycol of molecular weight 5 kD (mal-PEG). Each mal-PEG addition causes a decrease in migration in SDS-PAGE corresponding to about 15 kD of protein, consistent with all six cysteines in the Eps1p variant being modified. Exposure of the Trx2 CXXC cysteines to reduction by DTT thus correlated with reduction of the two CX6C structural disulfides in the Trx1 and Trx2 domains. (DOCX) [file pone.0113431.s002.docx]
